# Hinokitiol-Loaded Mesoporous Calcium Silicate Nanoparticles Induce Apoptotic Cell Death through Regulation of the Function of MDR1 in Lung Adenocarcinoma Cells

**DOI:** 10.3390/ma9050306

**Published:** 2016-04-25

**Authors:** Yu-Fang Shen, Chia-Che Ho, Ming-You Shie, Kan Wang, Hsin-Yuan Fang

**Affiliations:** 1 3D Printing Medical Research Center, China Medical University Hospital, Taichung City 40447, Taiwan; cherryuf@gmail.com (Y.-F.S.); sfox1223@gmail.com (C.-C.H.); eviltacasi@gmail.com (M.-Y.S.); 2 H. Milton Stewart School of Industrial and Systems Engineering, Georgia Institute of Technology, Atlanta, GA 30332, USA; kan.wang@gatech.edu; 3 Georgia Tech Manufacturing Institute, Georgia Institute of Technology, Atlanta, GA 30332, USA; 4 Department of Thoracic Surgery, China Medical University Hospital, Taichung City 40447, Taiwan; 5 School of Medicine, China Medical University, Taichung City 40447, Taiwan

**Keywords:** mesoporous calcium silicate, hinokitiol, apoptosis, multiple drug resistance protein 1, caspase-3/-9

## Abstract

Hinokitiol is a tropolone-related compound found in heartwood cupressaceous plants. Hinokitiol slows the growth of a variety of cancers through inhibition of cell proliferation. The low water solubility of hinokitiol leads to less bioavailability. This has been highlighted as a major limiting factor. In this study, mesoporous calcium silicate (MCS) nanoparticles, both pure and hinokitiol-loaded, were synthesized and their effects on A549 cells were analyzed. The results indicate that Hino-MCS nanoparticles induce apoptosis in higher concentration loads (>12.5 μg/mL) for A549 cells. Hino-MCS nanoparticles suppress gene and protein expression levels of multiple drug resistance protein 1 (MDR1). In addition, both the activity and the expression levels of caspase-3/-9 were measured in Hino-MCS nanoparticle-treated A549 cells. The Hino-MCS nanoparticles-triggered apoptosis was blocked by inhibitors of pan-caspase, caspase-3/-9, and antioxidant agents (N-acetylcysteine; NAC). The Hino-MCS nanoparticles enhance reactive oxygen species production and the protein expression levels of caspase-3/-9. Our data suggest that Hino-MCS nanoparticles trigger an intrinsic apoptotic pathway through regulating the function of MDR1 and the production of reactive oxygen species in A549 cells. Therefore, we believe that Hino-MCS nanoparticles may be efficacious in the treatment of drug-resistant human lung cancer in the future.

## 1. Introduction

Drug delivery is an important method for targeted tumor therapy, and the bioactivity of materials has great importance for promoting tissue regeneration [1,2,3,4]. Silica has been widely used for molecule immobilization. In most studies in this field, enzymes are entrapped in sol-gel silica matrices or mesoporous silica structures [5]. Several studies have proven that mesoporous silica (SiO_2_) nanoparticles may be used as drug delivery carriers with release kinetics that can be controlled by adjusting the internal and mesopores’ hollow microstructures [6,7,8]. Because of their enriched surface hydroxyl groups, the mesoporous SiO_2_ nanoparticle can easily be modified for further application, and have low toxicity for use in biomedical work [9]. Therefore, mesoporous SiO_2_ nanoparticles have been used for various bioengineering applications, such as intracellular molecule labeling, drug delivery, gene targeting, and diagnostic imaging [10,11,12].

Several research projects have verified that the degradation of mesoporous SiO_2_ nanoparticles is influenced by many factors, such as crystallinity, surface modification, particle morphology, and size [13,14]. However, the degradation of pure mesoporous SiO_2_ nanoparticles is inclined to be slow when compared to Ca-containing mesoporous SiO_2_ particles [15]. Several researchers have attempted to improve the technology for preparing mesoporous calcium silicate (MCS) particles with better physicochemical and biological behavior [16,17,18]. *In vitro* tests comparing micro- and nano-calcium silicate particles prepared using sol-gel processes have shown that the bioactivity, biocompatibility, and osteogenesis gene stimulatory effects of the sol-gel-derived nanoparticles are better than microparticles, probably due to their unique nanoscale structure and larger surface area [19]. Mesoporous bioglasses in both particle and scaffold form have been developed for tissue regeneration applications, revealing increased bioactivity as compared to pure mesoporous silica and conventional non-mesoporous bioglasses [20,21]. Of particular interest are the findings of Chen *et al.* [22] suggesting that the stimulation of proliferation and osteogenic-related protein synthesis by cultured primary cells after exposure to extracts of sol-gel calcium silicate particles are related to Si contact, but not to Ca, because no increased osteoblast activity was observed in the absence of Si ions. However, several other studies also indicate the role of Si in stimulating angiogenic differentiation *in vitro* [23].

Hinokitiol is a natural compound found in *Chamaecyparis Taiwanensis*, which can be culled from the wood of cupressaceous plants; it has diverse biological and pharmacological properties. Its biological properties include antiviral, antibacterial, antifungal, antitumor, and insecticidal tendencies, with negligible cytotoxicity [24,25]. In recent years, a growing body of research in several biomedical fields has shown the potential protective effects of hinokitiol against inflammation. It is capable of significant anti-inflammatory activity in a series of cells, managed by a variety of mechanisms [26]. In addition, the lung tumor suppressing abilities of hinokitiol occur without weight loss and result in low levels of toxicity in the host, indicating the potential of this naturally occurring compound as a therapeutic agent in lung cancer treatment [24]. The study also reported that hinokitiol can induce DNA damage and autophagy by cell cycle arrest and senescence in gefitinib-resistant lung adenocarcinoma cells [24].

To improve the bioavailability of hinokitiol, we designed and developed Hino-MCS (mesoporous calcium silicate nanoparticles loaded with hinokitiol). This study examined the molecular mechanisms triggered by Hino-MCS in the lung cancer cell line that was established in our laboratory and investigated the relationship between Hino-MCS concentration and the expression level of MDR1, which is correlated with chemo-resistance in lung cancer [27] and is an important protein of the cell membrane that pumps many foreign substances out of cells. The lung cancer cell line we used is unique in its resistance to cisplatin treatment and its ability to clarify the mechanism of Hino-MCS to promote bioavailability. The main goal of this study is to synthesize Hino-MCS using a sol-gel method and then examine its ability to deliver anti-cancer drugs as well as its functional effects on lung adenocarcinomas.

## 2. Materials and Methods

### 2.1. Synthesis and Characterization of Mesoporous Calcium Silicate Nanoparticles

Mesoporous calcium silicate (MCS) nanoparticles were prepared by a template method. Briefly, 3.3 g cetyltrimethylammonium bromide (CTAB, Sigma-Aldrich, St. Louis, MO, USA) and 6 mL NH_3_·H_2_O were mixed in double-distilled water (ddH_2_O, 300 mL) and stirred for 15 min at 60 °C. Next, 15 mL tetraethyl orthosilicate (TEOS) and 15.6 g calcium nitrate were added with vigorous stirring for 3 h. The precipitate products were collected by filtration and washed three times each with 1N hydrochloric acid and ethanol. After this, the collected powders were dried at 60 °C overnight and sintered at 550 °C for 2 h to remove remaining traces of CTAB. The nanoparticles were characterized by small-angle X-ray diffraction (Bruker D8 SSS, Karlsruhe, Germany) and transmission electron microscopy (TEM). The nanopore size distribution and nanopore volume were determined by Brunauer–Emmett–Teller (BET) analysis.

### 2.2. Hinokitiol Loading and Release from MCS Nanoparticles

The Hino-MCS nanoparticles were prepared by immersion in 100 mg of as-prepared MCS nanoparticles and hinokitiol (6.25–50 μg/mL, Sigma-Aldrich, St. Louis, MO, USA) of methanol, which was then shaken at room temperature for 24 h at room temperature. The Hino-MCS nanoparticles were then separated using centrifugation at 13,000 rpm for 20 min to remove unloaded hinokitiol and methanol. The Hino-MCS nanoparticles were then washed with ddH_2_O several times and dried at 37 °C for 24 h. The drug release kinetic of MSC nanoparticles was evaluated by soaking 10 mg of Hino-MCS nanoparticles in 1 mL of PBS (pH 7.4) at 37 °C. The PBS was removed for sampling and replaced with fresh PBS at various time points, and the amount of drugs released was determined using a UV-Vis spectrophotometer (TECAN Infinite Pro M200, Männedorf, Switzerland). The analysis was carried out in three separate groups of experiments. The standard curve was produced using PBS as the solvent with a correlation coefficient (R^2^) of 0.999.

### 2.3. Ion Concentration

After immersion for different time periods, the Ca, P, and Si ion concentrations released from Hino-MCS nanoparticles on PBS were analyzed using an inductively coupled plasma-atomic emission spectrometer (ICP-AES; Perkin-Elmer OPT 1MA 3000DV, Shelton, CT, USA). Three samples were measured for each data point. The results were obtained in triplicate from three separate samples for each test.

### 2.4. Cell Biocompatibility

Human adenocarcinoma alveolar basal epithelial cells (A549 cells; BCRC 60074, Hsin-Chu, Taiwan) were used throughout this study. Cell suspensions at a density of 5 × 10^3^ cells/well were directly seeded into a 96-well tray for 12 h and supplied Dulbecco’s modified Eagle medium (DMEM; Caisson Laboratories, North Logan, UT, USA) containing 10% fetal bovine serum (Caisson Laboratories, North Logan, UT, USA) and 1% penicillin (10,000 U/mL)/streptomycin (10,000 μg/mL) solution (Caisson Laboratories, North Logan, UT, USA) at 37 °C in a 5% CO_2_ atmosphere for 24 h. Afterward, 50 μg/mL of MCS nanoparticles in DMEM were added to the plate along with the MCS nanoparticles, and loaded with various concentrations of hinokitiol (0, 6.25, 12.5, 25, and 50 μg/mL). Cells cultured on a tissue culture plate were used as a control (Ctl). After being cultured until the end of the culture period, the medium was discarded and the wells were washed twice with cold PBS. Each well was then filled with the medium at a 1:9 ratio of PrestoBlue^®^ (Invitrogen, Grand Island, NY, USA) in fresh DMEM and incubated at 37 °C for 30 min, after which the solution in each well was transferred to a new 96-well plate. Plates were read in a multi-well spectrophotometer (TECAN Infinite Pro M200, Männedorf, Switzerland) at 570 nm with a reference wavelength of 600 nm. Cells cultured on the tissue culture plate without nanoparticles were used as a control (0 μg/mL). The results were obtained in triplicate from three separate experiments in terms of optical density (OD). The cell viability was normalized to the culture plate in terms of optical density.

### 2.5. Multiple Drug Resistance Protein 1 (MDR1) Gene and Protein Expression

The A549 cells at a density of 5 × 10^5^ in a 10-cm dish were incubated with or without Hino-MCS nanoparticles for 24 and 72 h. The cells were then collected and the total RNA of all groups was extracted using TRIzol reagent (Invitrogen) and analyzed using RT-qPCR. Total RNA (500 ng) was used for the synthesis of complementary DNA using a cDNA Synthesis Kit (GenedireX, Las Vegas City, NV, USA), following the manufacturer’s instructions. RT-qPCR primer was assessed for forward (GTGTGGTGAGTCAGGAACCTGTAT) and reverse (TCTCAATCTCATCCATGGTGACA) primers for MDR1 gene sequences from the NCBI Sequence database. SYBR Green qPCR Master Mix (Invitrogen) was used for detection, and the target mRNA expressions were assayed on an ABI Step One Plus real-time PCR system (Applied Biosystems, Foster City, CA, USA). Each sample was analyzed in triplicate.

In addition, the MDR1 protein of the A549 was analyzed using the Western blot process. The cells were lysed in an NP-40 buffer on ice for 1 h and the suspensions were centrifuged at 14,000 *g*. The cell lysates (40 μg protein) were separated using SDS-polyacrylamide gel electrophoresis and then transferred to nitrocellulose membranes. The membrane was blocked in 2% bovine serum albumin (BSA) for 30 min and then immunoblotted with the primary anti-MDR1 and β-actin (GeneTex, San Antonio, TX, USA) for 3 h, following which it was washed three times with a tris-base saline buffer containing 0.05% Tween-20. A horseradish peroxidase (HRP)-conjugated secondary antibody was subsequently added and the proteins were visually examined with enhancement using enhanced chemiluminescent detection kits (Invitrogen, Carlsbad, CA, USA). The stained band was then scanned and quantified using a densitometer (Syngene Bioimaging, Frederick, MD, USA) and ImageJ software (National Institutes of Health, Bethesda, MD, USA). The protein expression level was normalized to the β-actin for each group.

### 2.6. Assays for Caspase-3 and Caspase-9 Activities

The A549 cells were kept at a density of 2 × 10^5^ cells/well, and were put into 12-well trays and cultured with various concentrations of Hino-MCS nanoparticles for 24 h. Subsequently, the cells were harvested and the cell lysates (50 μg protein) assessed in accordance with the manufacturer’s instructions using the provided caspase-3 and caspase-9 colorimetric assay kits (Invitrogen, Carlsbad, CA, USA).

Cell viability was found to depend on the inhibition of caspase-3 and caspase-9 signaling, as pretreatment with its inhibitor (caspase-3 substrate: DEVD-pNA, or caspase-9 substrate: LEHD-pNA). After the pre-treatment described above, the cells were detached using trypsin-EDTA and seeded on a dish and treated with Hino-MCS nanoparticles (50 μg/mL) for 24 h. Cell viability was evaluated using the PrestoBlue^®^ assay (Thermo Fisher Scientific, Waltham, MA, USA). All experiments were carried out in triplicate.

### 2.7. Effects of the Reactive Oxygen Species (ROS) Scavenger on Cell Viability

A549 cells, at a density of 2 × 10^5^ cells/well, were placed into 12-well trays and pre-treated with N-acetyl-L-cysteine (NAC, Sigma, St. Louis, MO, USA), a ROS scavenger, for 2 h followed by treatment with or without 25 μg/mL and 50 μg/mL Hino-MCS nanoparticles. Cells were thereafter harvested at 24 h to investigate the percentage of viable cells, as elsewhere described.

### 2.8. Statistical Analysis

A one-way variance statistical analysis was used to evaluate the significance of the differences between the means in the measured data. Scheffe’s multiple comparison test was used to determine the significance of the deviations in the data for each specimen. In all cases, the results were considered statistically significant with *p* value < 0.05.

## 3. Results and Discussion

### 3.1. Characterization of MSC Nanoparticles

**Figure 1:**
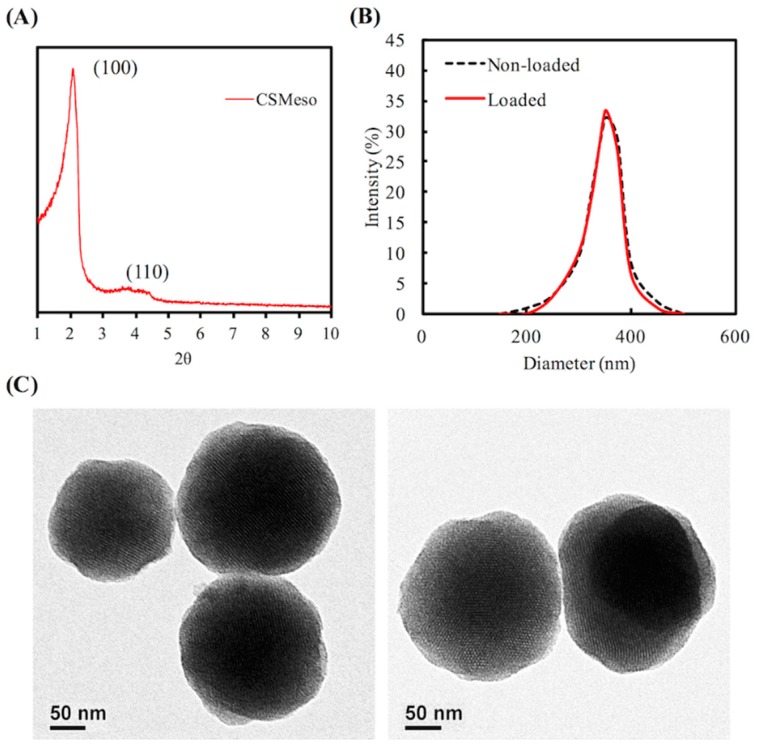
(**A**) Wide-angle XRD; (**B**) DLS; and (**C**) TEM analyses of MCS nanoparticles.

There were two obvious characteristic peaks, at 2θ = 2.5 and 4, in the small-angle XRD pattern (Figure 1A), indicating that the MCS nanoparticles had an orderly meso-structure. The MCS nanoparticles showed much better dispersibility (Figure 1B). The average particle sizes, determined by DLS for MCS nanoparticles and hinokitiol-loaded MCS nanoparticles, were 325 nm and 352 nm, respectively. Compared with Figure 1C, no apparent difference was found before (left) and after (right) hinokitiol loading in mesoporous MCS nanoparticles (Figure 1C). Besides, Gan’s group [28] reported mesoporous silica nanoparticles within 55–440 nm could all be internalized into the cells. Our average particle sizes of mesoporous calcium silicate nanoparticles and hinokitiol-loaded mesoporous calcium silicate nanoparticles are lower than 440 nm. Therefore, we suggested our mesoporous calcium silicate nanoparticles can be taken up into cells.

### 3.2. Hinokitiol Delivery from MBG Nanoparticles

**Figure 2:**
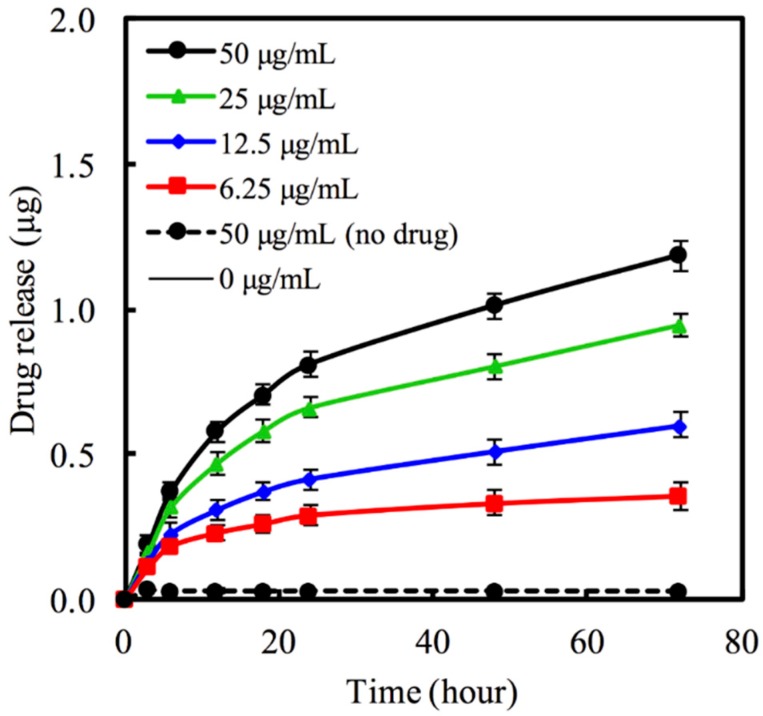
Release amount of hinokitiol from MCS nanoparticles in PBS (pH 7.4) at 37 °C.

The MCS nanoparticles carrying various concentration of 6.25–50 μg/mL hinokitiol in PBS was used to evaluate hinokitiol release. An *in vitro* hinokitiol release profile was obtained by considering the fraction of drug release in PBS (pH 7.4) with respect to Hino-MCS nanoparticles. Figure 2 shows the amount of hinokitiol released from MCS nanoparticles. The results indicate that hinokitiol was successfully loaded in the MCS nanoparticles. However, during the first 6 h the release behavior of the Hino-MCS nanoparticles showed little variation. All of the samples exhibited burst release for the loaded hinokitiol in the first 24 h, while the drug release for MCS nanoparticles sustained release continuously for 72 h (Figure 2). One of the most interesting results is that the prepared MCS nanoparticles possess a high hinokitiol-loading efficiency. However, the release kinetics of hinokitiol in MCS nanoparticles can be effectively controlled by adjusting the loaded drug concentrations. Previous studies have clearly shown that surface area, mesopores’ volume, and pore size play important roles in promoting drug-loading efficiency in mesoporous nanoparticles [10,29]. In this study, the surface area and pore volume of prepared MCS nanoparticles were higher than powders, and these may be the two main reasons for the increase in hinokitiol loading efficiency. The synergistic effect of hinokitiol can be applied in biomaterials to increase their antibacterial activity, also without cytotoxicity [30].

**Figure 3:**
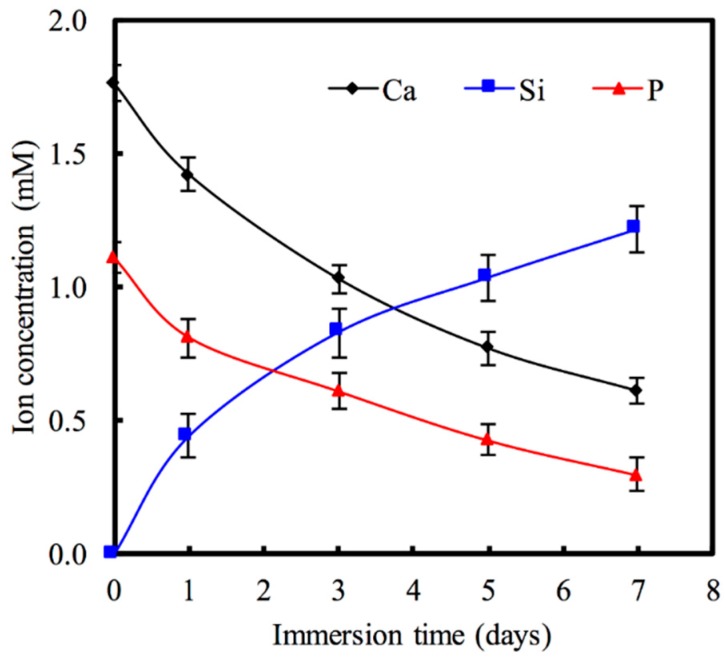
The change of Ca, Si, and P concentrations of PBS with MCS nanoparticles for various days.

The release of ions from MCS nanoparticles is also an important aspect of cell behavior. Variations in the SBF Ca, Si, and P ion concentrations after being immersed for various time periods are shown in Figure 3. The Ca ion concentration of the solution decreased to approximately 1.42 mM after one day, which is lower than the baseline Ca concentration (1.76 mM) of SBF (*p* < 0.05). A recent study suggests that loaded drugs may be chelated with Ca on the pore walls of MCS nanoparticles to increase loading efficiency and reduce the burst release of the drugs they carry [15]. Therefore, our study’s findings that Ca in MCS nanoparticles significantly increases the efficiency of drug-loading may prove to be of great importance. As for the P ion concentration of the solution, it decreases for all time points, whereas Si concentrations increase steadily with time. The Si ion concentrations of the solution were 0.44, 0.83, and 1.21 mM after having been immersed for 1, 3, and 5 days, respectively. Taking cell functions into account, the appropriate silicon ions being released from silicate-based materials may have a positive impact on cell behavior and functions [31,32]. Valerio *et al.* [33] report that the silicon ion concentration (<2 mM) from the dissolution of a bioglass not only enhances osteoblast proliferation, but also stimulates collagen production compared with biphasic calcium phosphate and the control. In addition, an appropriate Si ion concentration may promote cell proliferation by stimulating the entry of cells into the S and G2 phases of the cell cycle [34]. 

### 3.3. The Effect of Hinokitiol Delivery on A549 Viability

**Figure 4:**
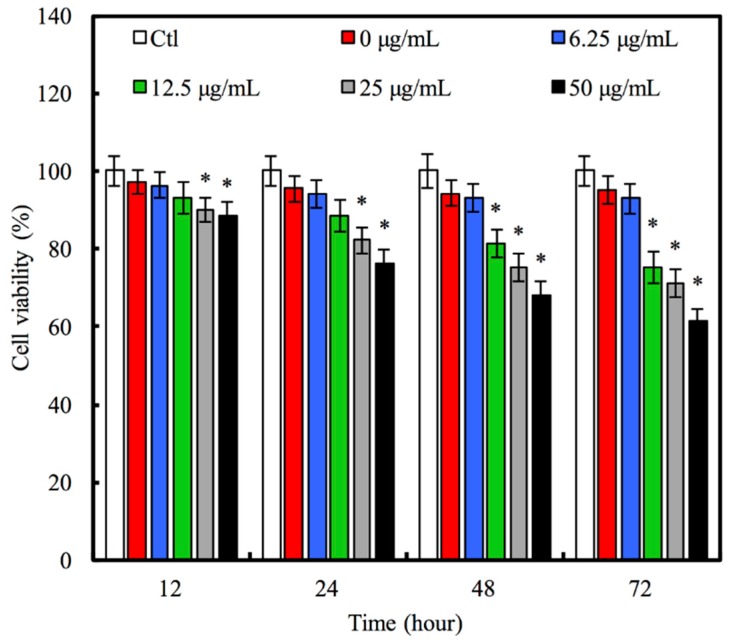
Cell viability of A549 cells after various time-points in culture with 50 μg/mL MCS nanoparticles loaded different concentration of hinokitiol. Star indicates a significant difference (*p* < 0.05) compared to 0 μg/mL.

No significant difference in A549 viability among MCS nanoparticles was detected after 12 h, and the hinokitiol concentration-loaded MCS nanoparticles (0, 6.25, and 12.5 μg/mL) was low (Figure 4). As the culture time increased, the viability of A549 in 0 μg/mL and 6.25 μg/mL Hino-MCS nanoparticles still supported the growth of A549 cells; however, at hour 24, the Hino-MCS nanoparticles in concentrations of 12.5–50 μg/mL showed a significant inhibitory effect on A549 viability, compared to MCS nanoparticles and Ctl (*p* < 0.05). At hour 72, the viability of A549 in Hino-MCS nanoparticles in concentrations of 12.5–50 μg/mL had been greatly inhibited (*p* < 0.05). At both hour 12 and 24, there was no significant difference in A549 cells’ viability among MCS nanoparticles and Hino-MCS nanoparticles in concentrations of 6.25 and 12.5 μg/mL, as well as Ctl. Hinokitiol is an essential plant-derived oil. It is a naturally occurring, aromatic, seven-membered tropolone compound. It has been reported to have applications in regulating several biological properties [24,25,35], and has also been shown to have inhibitory effects in several cancer cell lines [24,36,37]. In previous studies, the effective dose of hinokitiol against cancer cells ranged from 5 to 200 μM [37]. Considering the bioavailability and the inhibitory evidence found in previous research, in this study we initially used dosages with concentrations of 6.25 μg/mL, loaded in MCS nanoparticles. In addition, previous study [37] also reported that the 200 μM of hinokitiol has significant antimicrobial and cytotoxic activities against oral pathogens and oral squamous cell carcinoma cell lines, respectively. Furthermore, our data shows that the IC_50_ of Hino-MCS nanoparticles in A549 cells is more than 50 μg/mL (about 300 μM).

### 3.4. Hino-MCS Nanoparticles Induce Apoptosis and Suppress MDR1 in A549 Cells

**Figure 5:**
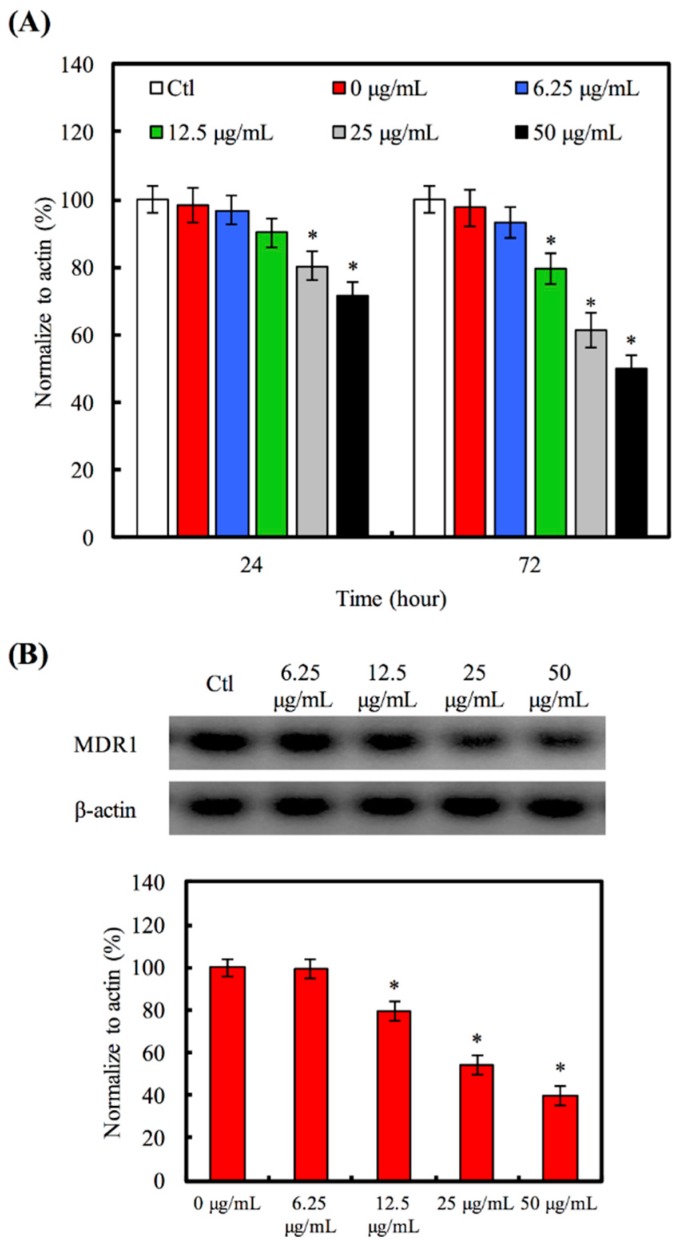
The MDR1 (**A**) gene and (**B**) protein expression of A549 cell cultured with various groups of Hino-MCS nanoparticles for three days. Star indicates a significant difference (*p* < 0.05) compared to 0 μg/mL.

We evaluated the MDR1 gene (Figure 5A) and protein (Figure 5B) in A549 cells treated with various concentrations (6.25–50 μg/mL) of Hino-MCS nanoparticles for 24 h. We also examined Hino-MCS nanoparticles at 25 and 50 μg/mL and found that after 24 h there was an inhibited level of MDR1 gene expression in the A549 cells. To determine whether MDR1 protein was stimulated when Hino-MCS nanoparticles were added to A549 cells, we quantified the activities of these proteins after 12 h; Western blot analysis demonstrated that after being cultured for 12 h the amount of MDR1 protein in A549 cells did not show noticeable differences between 0 and 6.25 μg/mL. In contrast to these findings, A549 cells cultured in the high concentration group (12.5–50 μg/mL) had been inhibited significantly, showing small amounts (*p* < 0.05) of MDR1 protein after 24 h compared to 0 μg/mL. Taken together, these results strongly suggest that the induction of A549 cells’ apoptosis had occurred, as well as suppression of multiple drug resistance by Hino-MCS nanoparticles. MDR1 overexpression is one of the most important reasons for multi-drug resistance to chemotherapeutic agents [38]. The results show that the mRNA and protein level of MDR1 expressions were both decreased in A549 cells after treatment with Hino-MCS nanoparticles. Therefore, the Hino-MCS nanoparticles decreased the interaction between drugs and MDR proteins in a dose-dependent manner. These results suggest that Hino-MCS nanoparticles induce A549 cell apoptosis via inhibiting the expression of MDR1 proteins by retaining more of the therapeutic medicine within the cells.

### 3.5. Hino-MCS Nanoparticles Trigger Intrinsic Apoptotic Cell Death in A549 Cells

**Figure 6:**
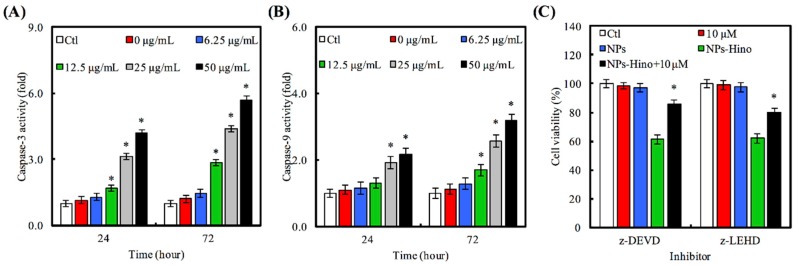
Effects of (**A**) caspase-3 and (**B**) caspase-9 activity on Hino-MCS nanoparticles-treated A549 cells. Star indicates a significant difference (*p* < 0.05) compared to 0 μg/mL; (**C**) Before A549 cells were exposed to Hino-MCS nanoparticles, they were pretreated with or without 10 μM of caspase-9 (z-LEHD) and caspase-3 inhibitor (z-DEVD), respectively. Star indicates a significant difference (*p* < 0.05) compared to cell without pretreatment inhibitor.

We will now address whether Hino-MCS nanoparticles induce apoptosis in A549 cells that were cultured with Hino-MCS nanoparticles for 24 h before being subjected to caspase-3/-9 activity. Our results show that Hino-MCS nanoparticles stimulate caspase-3 (Figure 6A) and caspase-9 (Figure 6B) activity after exposure for 24 h. In order to confirm the roles of caspase cascade-mediated apoptosis by Hino-MCS nanoparticles, we treated A549 cells both with and without z-DEVE (a caspase-3 inhibitor) and z-LEHD (a caspase-9 inhibitor) before exposing them to Hino-MCS nanoparticles to analyze their viability. Our results indicate that both caspase protease inhibitors (z-DEVD and z-LEHD) had great success in defending against Hino-MCS nanoparticle-triggered cell death, thus increasing the viability of A549 cells (Figure 6C). We feel that these findings can be seen as evidence that the intrinsic caspase contributes to Hino-MCS nanoparticles-induced cell death via apoptosis pathway in A549 cells, indicating that the Hino-MCS nanoparticles inhibit A549 cell growth and induce apoptotic cell death in direct proportion to both concentration and length of exposure. It seems reasonable to suggest that these effects occur through intrinsic signaling or mitochondrial-dependent pathways which are connected with the activation of caspase-9 and caspase-3. There is accumulating evidence indicating that mitochondria play an important role in the apoptotic process in human cells [39]. The early stages of apoptosis precede the efflux of various small molecules from the mitochondria (including cytochrome c, apoptosis-inducing factor, and cIAPs) and are followed by caspase-3/caspase-9 cascade activation [40]. Various studies have shown that hinokitiol induces apoptosis through intrinsic signaling pathways by depolarizing the mitochondrial membrane and triggering the release of cytochrome c into cytosol, followed by the cleavage of caspase-9/-3 [41]. We have found caspase-3 cleaved into Hino-MCS nanoparticle-treated A549 cells, which induced apoptosis by hinokitiol released from MCS nanoparticles. This shows great promise for the positive influence of the activation of caspase-3/-9 for therapeutic treatment of Hino-MCS nanoparticles for lung adenocarcinomas.

### 3.6. Hino-MCS Nanoparticles Enhance ROS-Generation-Dependent A549 Cell Apoptosis

**Figure 7:**
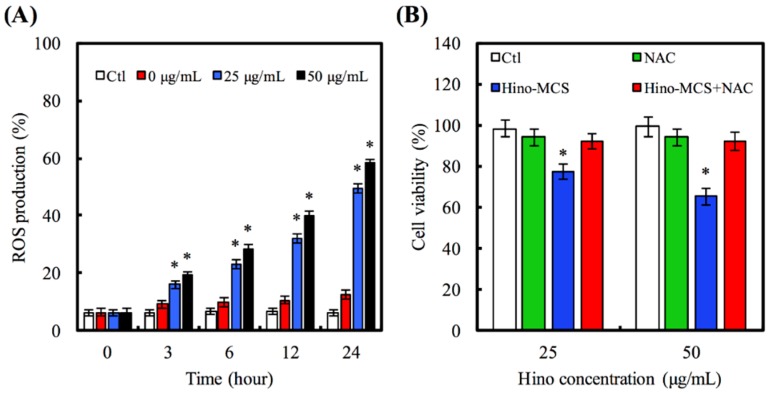
(**A**) A549 cells in response to 25 and 50 μg/mL Hino-MCS nanoparticles for various time points and the ROS production. Star indicates a significant difference (*p* < 0.05) compared to 0 μg/mL; (**B**) For cell viability, A549 cells were exposed to 25 and 50 μg/mL Hino-MCS nanoparticles for 48 h before pretreated with or without NAC, respectively. Star indicates a significant difference (*p* < 0.05) compared to cell with pretreatment inhibitor.

**Figure 8:**
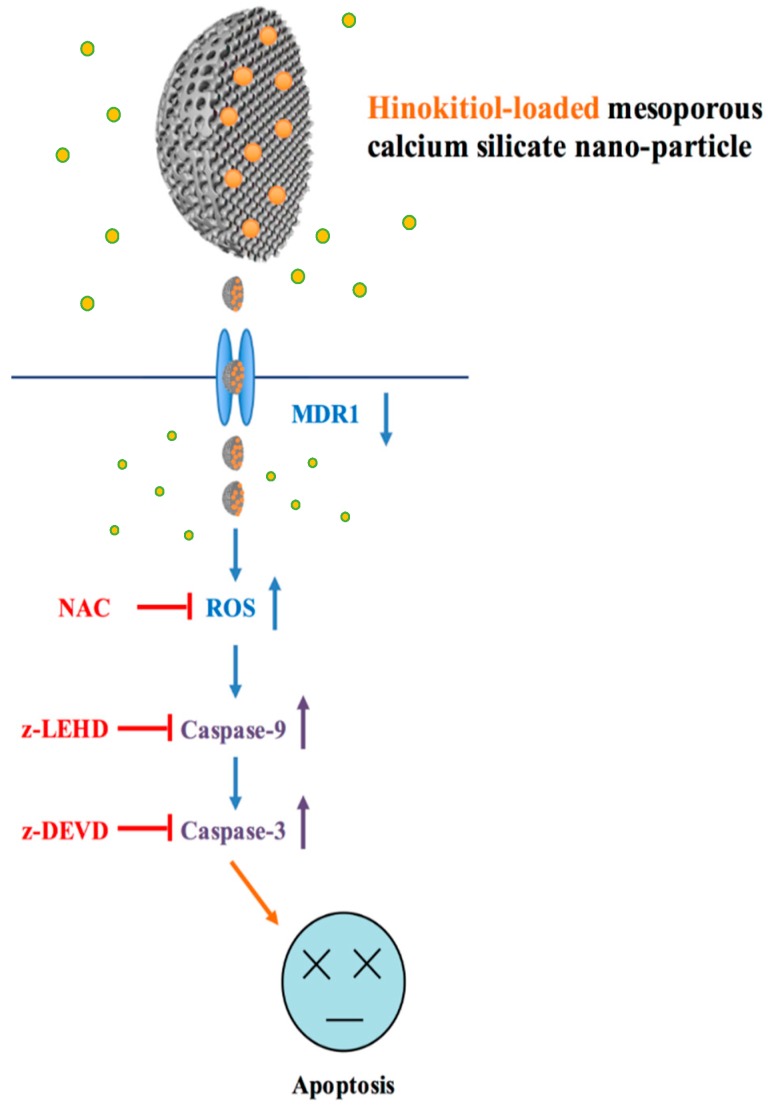
Schematic diagram of molecular mechanism by MCS nanoparticles loaded with hinokitiol (Hino-MCS nanoparticles) regulating the signaling pathway via MDR1 leading to apoptosis in human lung cancer cells.

We also made a close examination to determine whether oxidative stress regulates Hino-MCS nanoparticle-provoked cell death; our findings demonstrate that Hino-MCS nanoparticles increase ROS levels in A549 cells, as shown in Figure 7A. In ROS, 25 μg/mL and 50 μg/mL were markedly upregulated, causing the differences they exhibited to increase significantly by hour three: (*p* < 0.05) by 1.77 and 2.11 times compared with 0 μg/mL, respectively. In addition, Figure 7B shows that the presence of NAC dramatically protects A549 cells from cell death. A549 cell viability did not vary significantly between Ctl, NAC, and Hino-MCS nanoparticles with NAC (*p* > 0.05). We further examined the effects of Hino-MCS nanoparticles on mitochondria-dependent signaling in A549 cells and found that caspase-3 was cloven in Hino-MCS nanoparticle-treated A549 cells, which induces apoptosis through the release of hinokitiol from MCS nanoparticles. Meanwhile, substantial increases in ROS levels were also observed in the 25 μg/mL and 50 μg/mL groups, which induce apoptosis by releasing hinokitiol from MCS nanoparticles. Since A549 cells are essential components of the leukemic microenvironment in promoting leukemic cell survival and chemoresistance, we determined whether cells affected hinokitiol-induced apoptosis in A549 cells [42]. More viability in A549 cells was seen when the cells were pretreated with an antioxidant agent (N-acetylcysteine; NAC) compared to the non-treated group. These results suggest that Hino-MCS nanoparticle-induced apoptosis can be carried out through the ROS production (Figure 8).

## 4. Conclusions

Our results may have important clinical applications for patients with chemotherapy-resistant lung cancer. Hino-MCS nanoparticles induce cell apoptosis in A549 cells and inhibit cell growth in higher loaded hinokitiol groups. Firstly, a dose-dependent increase in lower cell viability was observed concomitantly with apoptotic changes in A549 lung cancer cells after Hino-MCS nanoparticle treatment. The findings suggest that Hino-MCS nanoparticles trigger apoptotic cell death by regulating the function of MDR1 and the production of ROS. The activation of caspase-9/-3 connected to intrinsic signaling pathways is the major pharmacologic action of Hino-MCS nanoparticles. Hino-MCS nanoparticles show promise for development as a novel medicine against drug-resistant human lung cancer.
